# Automatic Temporal Expectancy: A High-Density Event-Related Potential Study

**DOI:** 10.1371/journal.pone.0062896

**Published:** 2013-05-01

**Authors:** Giovanni Mento, Vincenza Tarantino, Michela Sarlo, Patrizia Silvia Bisiacchi

**Affiliations:** Department of General Psychology, University of Padua, Padua, Italy; Duke University, United States of America

## Abstract

How we compute time is not fully understood. Questions include whether an automatic brain mechanism is engaged in temporally regular environmental structure in order to anticipate events, and whether this can be dissociated from task-related processes, including response preparation, selection and execution. To investigate these issues, a passive temporal oddball task requiring neither time-based motor response nor explicit decision was specifically designed and delivered to participants during high-density, event-related potentials recording. Participants were presented with pairs of audiovisual stimuli (S1 and S2) interspersed with an Inter-Stimulus Interval (ISI) that was manipulated according to an oddball probabilistic distribution. In the standard condition (70% of trials), the ISI lasted 1,500 ms, while in the two alternative, deviant conditions (15% each), it lasted 2,500 and 3,000 ms. The passive over-exposition to the standard ISI drove participants to automatically and progressively create an implicit temporal expectation of S2 onset, reflected by the time course of the Contingent Negative Variation response, which always peaked in correspondence to the point of S2 maximum expectation and afterwards inverted in polarity towards the baseline. Brain source analysis of S1- and ISI-related ERP activity revealed activation of sensorial cortical areas and the supplementary motor area (SMA), respectively. In particular, since the SMA time course synchronised with standard ISI, we suggest that this area is the major cortical generator of the temporal CNV reflecting an automatic, action-independent mechanism underlying temporal expectancy.

## Introduction

Time processing is one of the most pervasive aspects of our mental functioning since it is involved in all motor, perceptual, and cognitive activities. Recently, there has been growing interest in understanding the cognitive mechanisms and the neural bases underlying timing [1]–[5]. A functional taxonomy of timing processes has been proposed by Coull and Nobre [6], which identifies distinct explicit and implicit mechanisms. According to the authors, explicit timing is engaged by tasks requiring either motor production (motor timing) or perceptual discrimination (perceptual timing). Conversely, implicit timing is indirectly engaged as an epiphenomenon of the temporal regularity of either a motor output (emergent timing) or a perceptual input (temporal expectation). Remarkably, temporal expectation may arise incidentally from a regular stimulus structure (exogenous temporal expectation) or may be consciously driven by informative pre-cues (endogenous temporal expectation).

Several functionally integrated brain structures have been found to be involved in time processing, including the cerebellum, the basal ganglia (BG), the insula, the dorsolateral prefrontal cortex (DLPFC), the inferior parietal cortices (IPC), the premotor cortex and the supplementary motor area (SMA) [5]–[10]. Remarkably, some of the above structures like the cerebellum, the BG, the SMA and the premotor cortex have traditionally been associated with motor processing, including action planning, response setting, preparation and selection [11]–[13]. This suggests that specific action-related processing may to some extent underlie, or at least share, brain circuits with perceptual timing mechanisms [14]–[17]. This hypothesis makes sense by virtue of the fact that accurate timing is of crucial importance for motor preparation and for limb-movement execution, even in the case in which a given action does not require an overt estimation of the passage of time. This is what the daily experience suggests when we implicitly calculate the appropriate time to cross the road immediately before the passage of a car. A wrong temporal control of the motor pattern would be really dangerous for our survival in the world. As such, it is reasonable to think that complex and dedicated sensorimotor circuits representing temporal dynamics with a high level of precision may be ubiquitously involved in activities requiring timing. Obviously, this would hold true either for complex goal-directed actions based upon high-level motor processing in time (e.g., playing a challenging Paganini’s solo on the violin) but also when motor demand is very low, as in the case of simple reaction to stimuli (e.g., pressing a button to signal onset).

Given its documented implication in motor processing, the SMA represents an ideal candidate for processing temporal aspects of movements. In fact, experimental evidence confirming the ubiquitous involvement of the SMA in both motor and perceptual timing has been brought by functional imaging studies [18]–[23], as recently reviewed in a wide voxel-wise meta-analysis by Wiener et al. [24]. What the exact role of this structure is in timing, however, is not fully understood yet. In an elegant explicit timing study, Coull et al. [18] found that even when motor task demand is kept very low, for example by using a time discrimination rather than reproduction task, a brain network including the SMA together with the DLPFC, the putamen and the right superior temporal gyrus (r-STG) was recruited. Importantly, the SMA was the only cortical region activated in the encoding and comparison phases of intervals. This led the authors to argue that this area plays a central role in the interval-tracking process underlying explicit perceptual timing rather than in time-based motor processing. Although to a lesser extent, the SMA has been proposed to play a key role also in implicit timing. In this regard, using an endogenous temporal expectation paradigm in which a central cue pre-oriented attention on time, Coull et al. [25] found that short and long SOAs between the cue and the target differentially engaged structures traditionally associated with motor preparation, with the SMA and the basal ganglia being significantly activated during long SOAs. In the authors’ view, the involvement of the SMA in the implicit orienting of attention on time raised the possibility of this area being associated with self-generated timing in addition to motor processes.

There is nonetheless no total agreement on the exclusive ‘perceptual’ role of the SMA in timing mechanisms. Indeed, the SMA has been shown to be activated only in temporal tasks with a strong motor component, such as time reproduction, but not in temporal perceptual tasks, arguing that different brain circuits serve motor and perceptual timing [26]. As well, in one experiment [27], the SMA has been selectively implicated with appropriate response selection rather than with the appropriate timing of the response under an uncertainty task condition.

As a critical consideration, it should be noted that most of the studies that investigated the neural bases of timing adopted motor tasks, such as interval production or reproduction, making it difficult to carve the neural bases of timing *per se* from additional task-related processes. The use of discrimination paradigms partially allows a by-pass of this problem by asking participants to delay the motor response after the comparison of an interval to a standard one, instead of reproducing it. However, even when motor preparation processes are minimized, discrimination paradigms may fail to unravel the contribution of further task requirements other than pure timing and motor preparation/execution, including response selection between alternatives, a process found to mainly involve the SMA [11]. As a consequence, it is difficult to disentangle between the neural activity more directly associated with timing by itself and that related to additional task-related processes. Hence, to what extent the SMA engagement in timing tasks reflects the contribution of non-perceptual, motor processes is still unknown.

### The Contingent Negative Variation as an Electrophysiological Index of Timing

Event-Related Potentials (ERPs) studies have also tried to shed light on the temporal dynamics of the brain activity underpinning timing [28]. Most of these studies investigated the Contingent Negative Variation (CNV). The CNV is a slow cortical ERP response of negative polarity reflecting both expectancy and motor preparatory processes that precede warned and to-be-responded imperative events [29]. Interestingly, CNV has also been consistently demonstrated to be a reliable electrophysiological hallmark of timing, since its morphological features, including peak and slope inversion latency, mirror the duration of a previously encoded target interval when it has to be processed in temporal reproduction, discrimination or bisection tasks [28], [30]–[37]. In line with the well-known Scalar Expectancy Theory (SET) proposed by Gibbon et al. [38], one main interpretation of the temporal CNV is that it reflects a time-unit ‘accumulator’ mechanism [30]–[32]. This hypothesis has arisen from the observation that the longer the estimated duration, the larger the CNV over the SMAs. However, the CNV ‘cumulative’ account has been recently challenged by Kononowicz et al. [39] who failed to replicate CNV amplitude modulation in relation to performance-based variations in a temporal reproduction task as originally reported [30]–[32]. Thus, there is evidence that the CNV may reflect a more indirect mechanism in timing than a pure direct reflection of the accumulation of pulses.

In addition to its functional role, it is noteworthy that the CNV has been reliably elicited both in explicit [30]–[34] as well as in implicit timing tasks [40] supporting the hypothesis that timing properties of slow brain potentials are transversal in temporal processing. It should be noted, however, that although similar in morphology, the implicit and explicit temporal CNV show different scalp distributions, presumably due to distinct cortical sources of activation. In explicit timing, the SMA would be the most probable candidate as the CNV generator [30]–[34]. The SMA recruitment in explicit tasks may reflect the cortical level of a wider dopaminergic striato-frontal pathway connecting the basal ganglia to the prefrontal areas via the thalamus, a network known to have a major role in temporal processing [5], [41]–[46]. In contrast, the implicit CNV seems to predominantly originate from activation of the bilateral premotor cortex [39]. One main explanation linking the temporal CNV with premotor area activation consists of the fact that event predictability may trigger anticipatory, action-directed neural activity resulting in optimization of behaviour, like for example, faster reaction times. Taken together, however, available fMRI and EEG data do not allow the complete exclusion of the possibility that the temporal CNV may, to some extent, be contaminated by task-related ERP activity not directly associated with timing mechanisms. This ultimately raises the possibility that the CNV can emerge from the overlapping of the activity of different neural structures associated with motor and/or perceptual processes, depending on the task requirements.

The aim of the present study was to unravel the contribution of task-related processes, including response preparation, selection and execution, from that more directly linked with timing mechanisms in a temporal expectancy task. To this purpose, we created a ‘passive temporal oddball task’, which was specifically designed to elicit temporal, expectancy-related CNV in the absence of task requirements. Participants were presented with pairs of stimuli (S1-S2), interspersed by a variable Inter-Stimulus Interval (ISI), which was manipulated according to an oddball probabilistic distribution. In 70% of trials, the ISI lasted 1,500 ms (1500-Standard ISI); in 15% of trials the ISI lasted 2,500 ms (2500-Deviant ISI); and in the remaining 15% of trials, the ISI lasted 3,000 ms (3000-Deviant ISI). Participants were told only to fixate on the centre of the screen; they did not receive any further instruction. As the main hypothesis, we speculated that if temporal expectancy were automatically arisen, then participants would have shown to be sensitive to the temporal regularities of sensory events even in the absence of any instruction or response associated to them. More specifically, the high probability that S2 would occur 1,500 ms after S1 offset would create an implicit temporal rule consisting of the maximum expectation that S2 would occur at that specific time-point. Consequently, we hypothesised that participant brain activity would become attuned to standard ISI, this being reflected in the elicitation of ERP responses correlated to the standard ISI duration. According to previous findings [30]–[37], we expected to find a shift in S1-locked CNV towards positivity (i.e., a CNV polarity inversion) during any intervals longer than the standard, at a point in time at which the comparison with the standard interval takes place. This should correspond in the present study to the time-point at which S2 had the highest probability of appearing, that is at the end of the standard ISI that we defined here as *S2 Maximum Expectation Time Point* or *S2 METP*. Moreover, in order to better understand the role of the SMA in automatic temporal expectancy as being dissociable from motor processes, we estimated the neural generators of both S1- and ISI-related ERP activity by using a 128-sensor, high-density array EEG system allowing a more reliable brain source analysis as compared to conventional EEG systems.

## Method

### Participants

Eighteen subjects (16 females; mean age = 24.6±4.5 years), recruited from the School of Psychology at the University of Padua, took part in the study. They received course credits for their participation in the study. All but three participants were right-handed according to the Edinburg Handedness inventory [47]. They had no history of neurologic, neuropsychiatric disorders, or drug consumption, and had normal or corrected-to-normal visual acuity. After being informed of the conditions of the study, all participants signed a voluntary consent form, and they were told that they could stop participating in the current experiment at any time. The study was approved by the ethical committee of Psychology of the University of Padua (protocol No. 1179) and was conducted according to the principles expressed in the Declaration of Helsinki.

### Experimental Procedure and Stimuli

**Figure 1:**
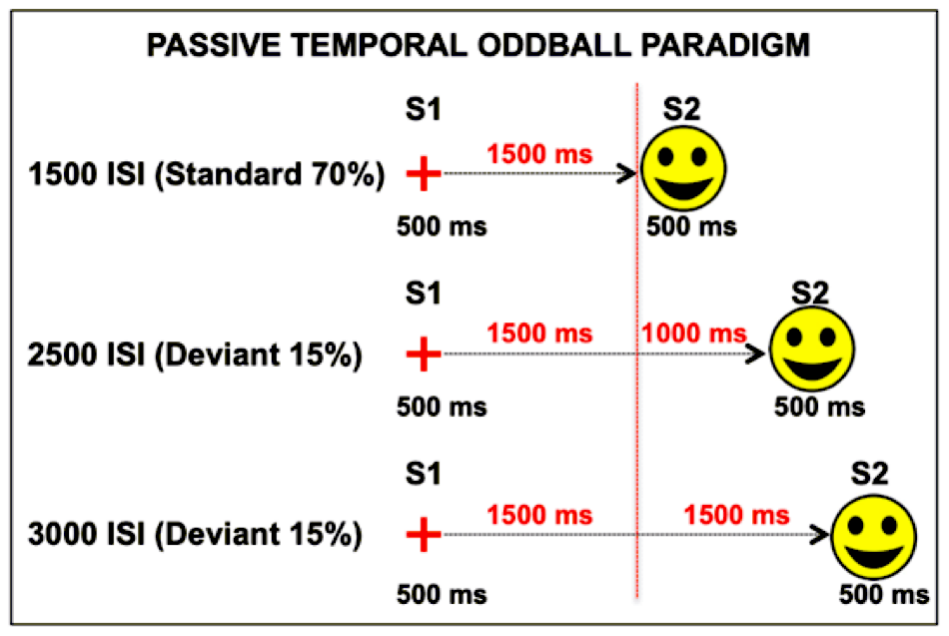
Passive temporal oddball task. S1 and S2 never changed across conditions. The only manipulated variable was the ISI length, which was 1,500, 2,500 and 3,000 ms long in the 1500-Standard ISI (70%), 2500-Deviant ISI (15%) and 3000-Deviant ISI (15%) conditions respectively. Participants were given neither instruction nor motor task. The vertical dotted red line represents the S2 maximum expectation time-point (S2 METP) corresponding to the end of the standard ISI.

The experiment was run in a dimly illuminated and electrically shielded room. Stimuli were presented on a 19-inch monitor at a resolution of 1280×1024 pixels. Participants were seated comfortably in a chair at a viewing distance 100 cm from the monitor. Stimuli consisted of two pictures consecutively presented in the centre of the screen (Fig. 1). The first stimulus (S1) consisted of a red cross (66×66 pixel, 72 dpi, 1.39°×1.29° of visual angle) presented for 500 ms, and the second stimulus (S2) was a picture of a yellow ‘smile’ surrounded by a black line (339×339 pixel, 72 dpi; 6.82°×6.63° of visual angle). Simultaneously to the presentation of S1, a 500 Hz simple sinusoidal tone was delivered, while the presentation of S2 was associated with a 1,000 Hz simple sinusoidal tone. Both acoustic stimuli lasted the same amount of time as the pictures (500 ms), and they were strictly synchronised in order to signal the beginning and end of each trial. The S1–S2 ISI was manipulated in order to create three different interval conditions, with different percentages of occurrence. In the first case, the 1500-Standard ISI, there was a 1,500 ms interval from S1 offset to S2 onset, whereas in the remaining two alternative conditions, i.e., the 2500- and 3000-Deviant ISI, it was lengthened to 2,500 and 3,000 ms, respectively. The percentage of occurrence was 70% for 1500-Standard ISI (210 trials), 15% for 2500-Deviant ISI (45 trials), and 15% for 3000-Deviant ISI (45 trials). The Inter-Trial-Interval (ITI) was randomly manipulated between 1,500 and 2,000 ms by using small 16-ms steps. The three conditions were randomly mixed and delivered in three separate 100-trial-blocks for a total of 300 trials, the only constraints were that two deviant trials could not be consecutively delivered and that at least two standard trials had to occur before a deviant one was presented. E-prime 2 software (Psychology Software Tools) was used to create and administer the stimuli. Participants were told only to fixate on the centre of the screen; they did not receive any further instruction.

### EEG Recordings

During the entire passive task, the EEG was continuously recorded using a Geodesic high-density EEG System (EGI GES-300), through a pre-cabled 128-channel HydroCel Geodesic Sensor Net (HCGSN-128) and referenced to the vertex. Scalp voltages were recorded and amplified during the entire experiment. The sampling rate was 500 Hz. The impedance was kept below 30 kΩ for each sensor. In order to reduce the presence of EOG artefacts, subjects were instructed to limit both eye-blinks and eye-movements as much as possible.

### ERP Analysis

ERP analyses were performed using Net Station 4.1 software (EGI®). To analyse the effect of ISI manipulation on CNV response, the EEG was segmented off-line into epochs starting 200 ms before S1 onset and ending 4,200 ms after it to allow coverage of the whole S1–S2 interval. Epochs were 20 Hz digitally low-pass filtered, and automatically processed to mark bad channels, which were identified as those containing EEG activity exceeding a threshold of 80 µV (min to max). Trials contaminated by eye-blink with a 120-µV threshold (min to max) or by eye-movements with a 55-µV threshold (min to max) were also marked by means of an automatic procedure implemented in the analysis software. In addition, all trials with more than 20% bad channels were automatically identified. Bad channels with more than 20% of rejected trials were interpolated with the surrounding electrodes. All the trials marked as bad were then manually inspected and rejected. The remaining EEG epochs contaminated by eye-blinks were corrected using Gratton’s algorithm [48] and re-referenced to the average of all electrodes. The artefact-free trials were then averaged for each participant, separately for each of the three conditions. The signal was aligned to the baseline by subtracting the mean signal amplitude in the pre-stimulus interval, i.e., from −200 to 0 ms relative to S1 onset. Two-dimensional reconstructions of scalp voltage were computed using a high-density, spherical spline interpolation map for each condition. Event-related potential amplitudes were analysed by pooling the values of six neighbouring electrodes within five regions of interest at a scalp-level (scalp-ROIs) identified on the basis of the 2D spline voltage maps. Five scalp-ROIs were calculated by averaging together six neighbour electrodes at the left-anterior (electrodes 12, 18, 19, 20, 22, 23, 24); right-anterior (electrodes 3, 4 5, 9, 10, 118, 124); central (62, 67, 71, 72, 76, 77); left-posterior (electrodes 50, 51, 52, 55, 58, 64) and right-posterior (electrodes 90, 91, 92, 95, 96, 97, 101). Two participants were excluded from ERP analysis due to their low number of artefact-free epochs.

### Statistical Analyses

According to the experimental hypothesis, S2 METP corresponds to the point at which the CNV should reach the highest value in all conditions before inverting towards baseline. To test this hypothesis, three regression equations (one for each condition) were fitted on the CNV signal for a time window (TW) of 800 ms immediately preceding S2 METP, called ‘TW-1’ (700 to 1,500 ms after S1 offset). We also extracted an additional 800-ms-long interval from 1,500 to 2,300 ms from S1 offset, which we called ‘TW-2’. Both the mean voltage amplitude and the slope of the CNV signal fitting TW-1 and TW-2 were extracted and compared by separated repeated measure ANOVA with ISI (1500-Standard *vs.* 2500-Deviant *vs.* 3000-Deviant ISI) and TW (TW-1 *vs*. TW-2) as within-subjects factors. Given that the conditions were associated with different numbers of trials, according to the literature [49]–[52] we ran all statistical analyses considering both the whole dataset (mean number of accepted trials for each ISI condition = 128.7±36.2, 34.11±5.4 and 36.9±6.2 for 1500-Standard, 2500-Deviant, and 3000-Deviant ISI, respectively) as well as a randomly extracted subset of data with an equal number of trials for each condition and participant (36.2±4.2, 34.11±5.4 and 36.9±6.2 for 1500-Standard, 2500-Deviant, and 3000-Deviant ISI, respectively).

Furthermore, since habituation effects on temporal-CNV have been previously reported [39], we run additional analyses to explore the presence of time-on-task effects on CNV amplitude and slope across the experimental session. To this purpose, we grouped the whole dataset trials in three consecutive blocks. This was done only for standard ISIs, since the number of trials for deviant ISIs was too low for a block-based clustering. Mean CNV amplitudes and slopes for TW-1 for each consecutive block and participant were entered in a repeated measure ANOVA with time-on-task (first *vs.* second *vs.* third experimental-block) as the main factor. For all ANOVAs the Bonferroni correction for multiple post hoc comparisons was applied when required. The ηp^2^s are provided for the qualitative comparison of effect sizes. All statistics were calculated with the software SPSS 18.0. The statistic results for both the whole dataset and the random data subset are reported. Single subject data for CNV amplitude and slope analyses are provided in Tab. S1.

### Brain Source Analysis

The cortical generators of S1-locked ERP activity were estimated. To do this, the conductive head volume was modelled according to the 3-spheres BERG method [53], as implemented in the Brainstorm software package [54], which is documented and freely available online for download under the GNU general public license (http://neuroimage.usc.edu/brainstorm). This method uses a spherical approximation of the head based on the creation of three concentric spheres with different homogeneous conductivity, representing the best-fitting spheres of the inner skull, outer skull, and scalp compartments extracted from the Montreal Neurological Institute (MNI) atlas. The solution space was constrained to the cerebral cortex, which was modelled as a three-dimensional grid of 15,028 fixed dipoles oriented normally to the cortical surface. The inverse transformation was then applied to the MNI canonical mesh of the cortex to approximate real anatomy. The EEG sensor positions were co-registered with the default anatomical mesh by employing rigid rotations and translations of digitized landmarks (anterior and posterior commissure, inter-hemispheric scissure, nasion, left and right tragus). The inverse problem was solved by applying the sLORETA [55]–[56] method implemented as a routine of the Brainstorm platform, which allows an estimation of the distribution of electrical sources in the brain. The covariance matrix was assumed to be independent across EEG sensors, with fixed variance computed from pre-stimulus recordings. For each participant, the sources were projected to a standard anatomical template (MNI) and their activity transformed in absolute Z scores relative to the baseline. The absolute values of the Z scores were then averaged across subjects. To examine which cortical regions were significantly activated, we entered the S1-locked source activation values into paired t-tests against the baseline. Thresholding on the size of the effects was applied: only clusters of at least 10 cortical vertices in the distributed sources model were considered. Then, the source map vertices where the *t* statistics exceeded a critical value (*P*<0.001; Bonferroni-corrected for multiple comparisons) were clustered into cortical regions of interest (cortical-ROI) based on their adjacency across the two-dimensional cortical sheet. Only the cortical-ROIs significantly activated were reported and identified according to the MNI coordinate system. Brodmann areas (BA) associated with the stereotaxic coordinates were also reported. Cortical map activations and statistics have been reported separately for two time windows, namely covering S1- (0 to 500 ms from S1 onset) and ISI-related (500 to 3,000 ms from S1 onset) activity. In order to more accurately depict the time course of the activation of the main cortical-ROIs identified, we used the scout analysis tool in Brainstorm. This procedure allows one to cluster subsets of neighbouring vertices and to plot their activation values for the temporal dimension.

## Results

**Figure 2:**
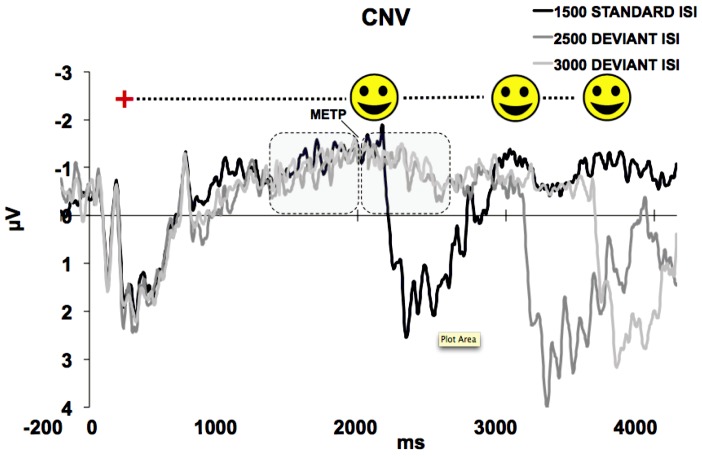
Grand Average of S1-locked ERP activity for Central scalp-ROI. The CNV showed a maximum amplitude value peaking at S2 METP, corresponding to around 2,000 ms from stimulus onset, that is, S1 (500 ms)+Standard ISI (1,500 ms).

Figure 2 displays the ERP grand average at central scalp-ROI, which showed the clearest CNV pattern. On the first morphological inspection, there were no differences among ISI conditions for the S1-related ERP activity. This time-window was characterised by the presence of a sensory-evoked ERP pattern showing a first negative peak at around 110 ms, a second positive peak at around 170 ms, and a third negative peak at around 250 ms followed by a large positive response covering the S1 duration and culminating in a sharp negative peak at around 1,000 ms from S1 onset. We then observed a sustained mounting negative signal corresponding to a CNV. Such a wave showed the same morphology and amplitude in all conditions and peaked at around 1,500 ms from S1 offset. This latency corresponded to the time-point at which the S2 onset was maximally expected (S2 METP) according to the standard ISI. After this point, in the standard ISI condition S2 appeared eliciting a large sensory-evoked activity. In contrast, in both the deviant ISI conditions the CNV showed a slope inversion turning toward the baseline, although S2 actually occurred here after several hundreds of milliseconds from S2 METP.

### CNV Amplitude

**Figure 3:**
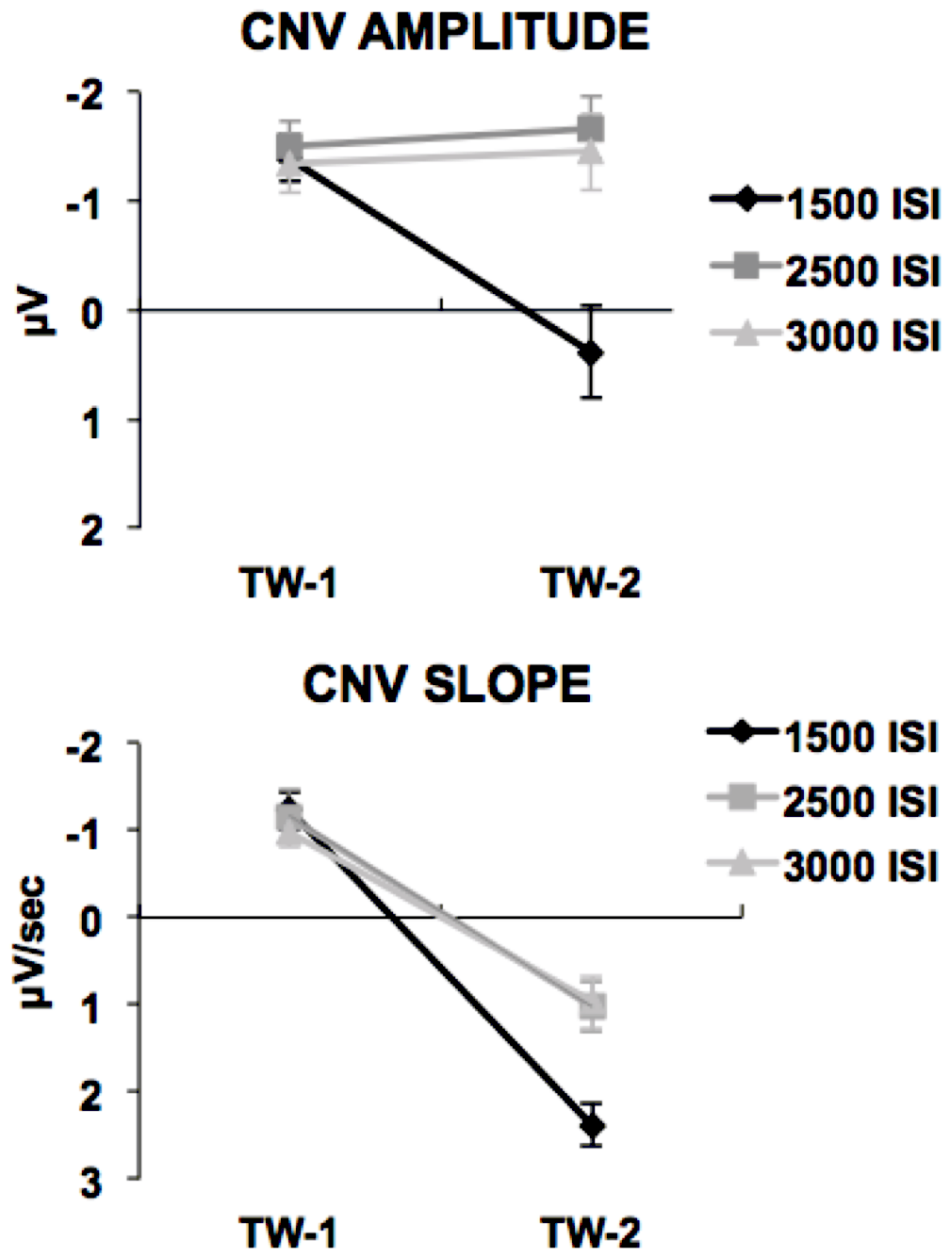
TW-1 *vs*.TW-2 CNV comparison for amplitude (3a) and slope (3b). The three ISI conditions showed the same pattern in TW-1 (i.e., before S2 METP) characterised by identical amplitudes and negative slopes. By contrast, in TW-2 (i.e., after S2 METP) the two deviant ISI conditions had equal amplitudes but positive slopes, while only the 1500-Standard ISI condition displayed larger amplitude due to S2 presentation. Bars represent standard errors.

The ANOVA performed on the CNV mean voltage amplitude on the randomly extracted subset of data yielded a significant main effect of ISI (F_(2,30)_ = 14.10; *p*<0.001; ηp^2^ = 0.48) and TW (F_(1,15)_ = 6.21; *p*<0.05; ηp^2^ = 0.29). As can be seen in Figure 3a, both the ISI and the TW effects were due to the fact that, only in TW-2, the ERP activity elicited by standard ISIs showed more positive amplitudes than when elicited by deviant conditions. This interpretation was confirmed by the ISI×TW interaction (F_(2,30)_ = 22.73; *p*<0.001; ηp^2^ = 0.6). Post-hoc comparisons revealed that in TW-1 the mean voltage amplitude did not differ among ISI conditions (*p*>0.9). Conversely, TW-2 standard ISI-related activity showed a larger, positive amplitude as compared to the two deviant conditions (*p*<0.01), although the latter two did not differ from each other (*p*>0.7). This effect was clearly due to the fact that, as previously mentioned, only for standard ISI the S2 METP was actually followed by S2 onset, resulting in a large predominantly positive ERP activity in TW-2. The ANOVA performed on the whole dataset including all the artefact-free trials for the 1500-Standard ISI condition yielded a main effect of ISI (F_(2,30)_ = 15.04; *p*<0.001; ηp^2^ = 0.5) and TW (F_(1,15)_ = 10.39; *p*<0.01; ηp^2^ = 0.40) as well as an ISI×TW interaction (F_(2,30)_ = 34.48; *p*<0.001; ηp^2^ = 0.69), confirming that the CNV amplitude across ISI conditions and time windows was not affected by the number of trials averaged.

### CNV Slope

The ANOVA performed on the beta values of the regression equations fitting the CNV signal in TW-1 and TW-2 for all ISI conditions on the randomly extracted subset of data yielded a main effect of ISI (F_(2,30)_ = 3.38; *p*<0.05; ηp^2^ = 0.18) and TW (F_(1,15)_ = 138.6; *p*<0.001; ηp^2^ = 0.9) as well as a ISI×TW interaction (F_(2,30)_ = 6.23; *p*<0.01; ηp^2^ = 0.29). As can be seen in Fig. 3b, post-hoc tests confirmed that, while in TW-1 the CNV slope displayed an equal, negative beta for all conditions (p = 1), by contrast in TW-2 the slope became positive for all conditions, although showing bigger values for the 1500-Standard ISI than for the 2500- and 3000-Deviant ISI (p<0.02), with the latter two not differing each other (p = 1). As for CNV amplitudes, this finding can be easily explained by the large ERP activity elicited by S2 for standard trials, which caused the slope to shift toward more positive values than for deviant trials. When considering the whole dataset instead of the randomly extracted data subset, the ANOVA highlighted again a main effect of ISI ISI (F_(2,30_ = 4.01; *p*<0.05; ηp^2^ = 0.21) and TW (F_(1,15)_ = 67.55; *p*<0.001; ηp^2^ = 0.81) as well as a ISI×TW interaction (F_(2,30)_ = 6.12; *p*<0.01; ηp^2^ = 0.29), meaning that, as for CNV amplitude analyses, the CNV slope was not affected by the number of trials averaged.

### Time-on-Task Effects

**Figure 4:**
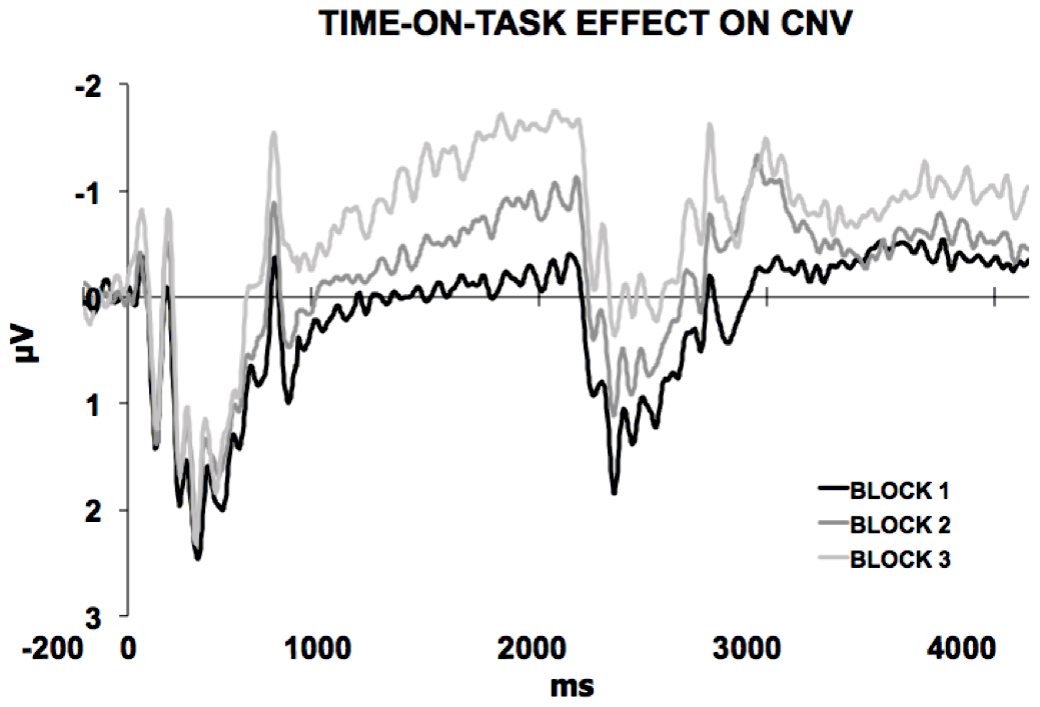
Time-on-task effect. The ERP activity plotted separately for the first, second and third block for the 1500-Standard condition revealed a time-on-task effect with the CNV showing more negative amplitude and slope as time passed by.

**Figure 5:**
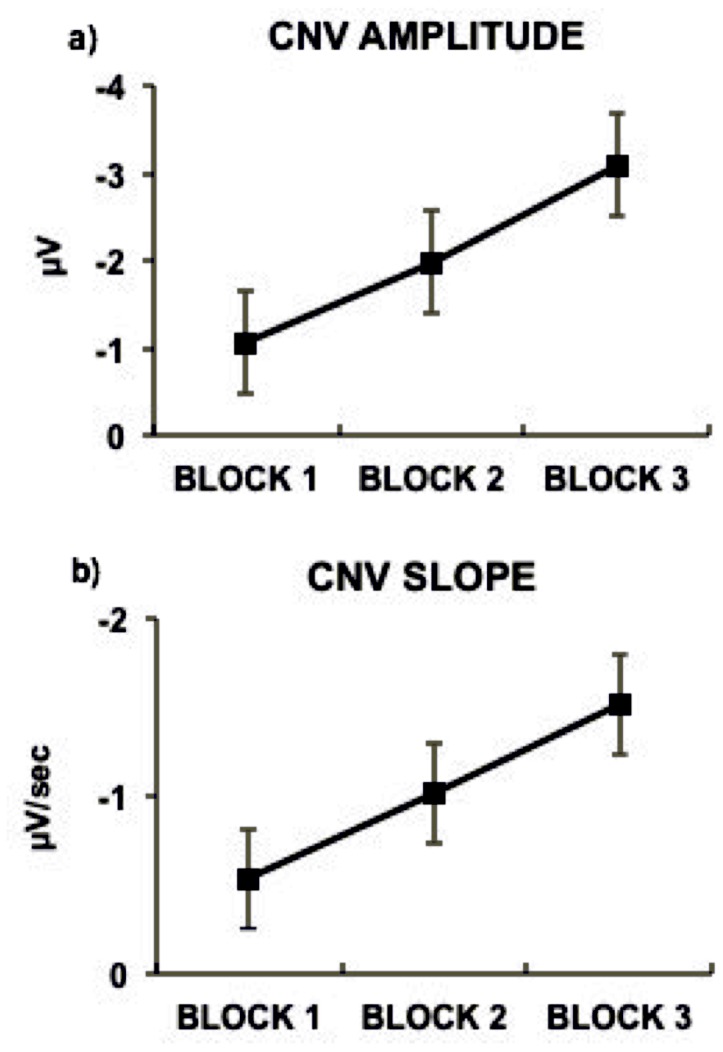
Block comparison for CNV amplitude and slope. Mean CNV amplitude (5a) and slope (5b) values for the first, second and third experimental block. Bars represent standard errors.

As shown in Fig. 4, the CNV became steeper and larger block-by-block while the S1-locked ERP activity did not differ as time passed. This phenomenon was confirmed by the ANOVA run on the mean CNV voltage, that showed a significant time-on-task effect consisting in the increasing of the amplitude of the CNV through the experimental session (F_(2,30)_ = 19.21; p<0.001; ηp^2^ = 0.56) Post-hoc test revealed that in the third experimental-block the CNV presented larger amplitude than for the second experimental-block (p<0.02). As well, the second experimental-block was characterized by larger CNV amplitude as compared to the first experimental-block (p<0.01) The time-on-task effect for CNV slope is shown in Fig. 5a. Analogously, a separate ANOVA yielded a significant time-on-task effect for CNV slope (F_(2,30)_ = 5.39; p<0.02; ηp^2^ = 0.26). Post-hoc tests revealed that the CNV was steeper in the third than in the second experimental-block (p<0.05) and in the second than in the first experimental-block (p<0.01). The time-on-task effect for CNV slope is shown in Fig. 5b.

### Brain Source Analysis Results

The sLORETA solution applied within the Brainstorm platform to S1-locked electrophysiological activity allowed us to identify the cortical generators of two time windows, that is, the S1-related ERP activity, including the sensory-evoked potentials occurring within the first hundreds of ms and the late, sustained ISI-related ERP activity, corresponding to the CNV. Since the comparison of the CNV amplitude and slope between the 2500- and 3000 deviant ISIs did not yield significant differences for the two time-windows considered, these two conditions were collapsed by calculating the average of their absolute cortical source values.

### S1-related Activity

**Figure 6:**
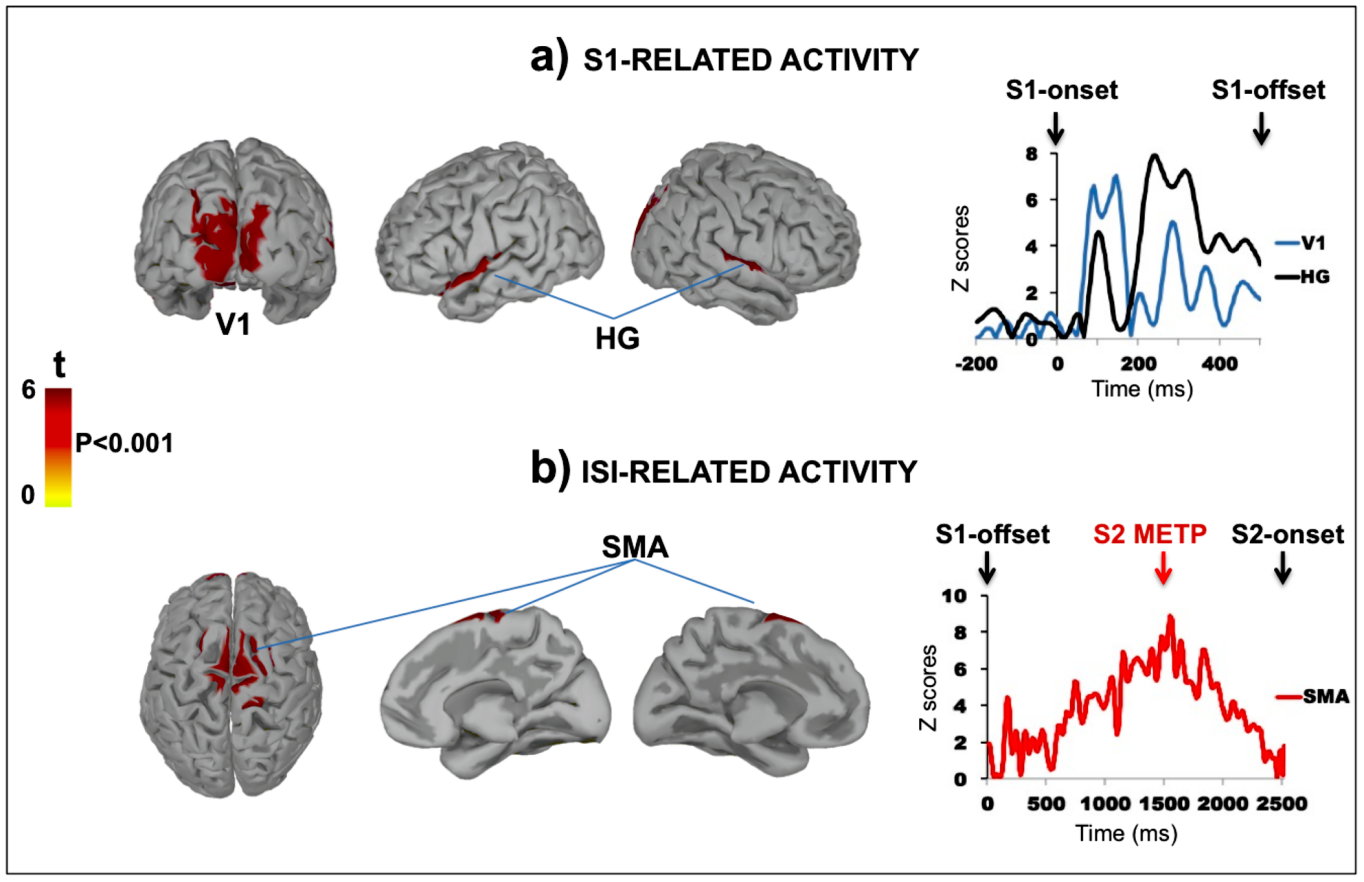
S1- and ISI-related brain-source analysis. a) *Leftward* *:* Cortical sources activation presented in Z scores according to the baseline and projected on a smoothed standard brain. The maps represent a 100–300 ms time-window from S1 presentation. Both visual (V1) and auditory (HG) primary areas showed significant activity (*p*<0.001; Bonferroni corrected); *Rightward:* time course activation of V1 and HG. The y-axis represents the mean standardised activation of adjacent vertices clustered within each significant cortical-ROI. The x-axis represents the entire temporal window considered for statistical analysis (0 to 500 ms from S1 onset); b) *Leftward:* ISI-related cortical sources activation at 1,500 ms from S1 offset, corresponding to S2 METP. Only the SMA showed a significant activity (*p*<0.001; Bonferroni corrected) starting at about 1,000 from S1 offset until S2 presentation. *Rightward:* time course activation of the SMA. The y-axis represents the mean standardised activation of adjacent vertices clustered within significant cortical-ROIs. The x-axis represents the entire temporal window considered for statistical analysis (0 to 2500 ms from S1 offset).

As shown in Figure 6, sLORETA yielded a bilateral occipital source for both standard and deviant conditions between 100 and 500 ms from S1 onset, corresponding to the negative-positive-negative-positive ERP complex observed in response to S1 onset. This activity was localised in the occipital areas corresponding to the left (BA 17; number of clustered vertices = 30; peak maximum x = −4.98, y = −110.81, z = 0.51; t>5) and right visual primary cortex (BA 17; number of clustered vertices = 31; x = 10.32, y = −104.03, z = −2.17; t>4). As expected because of the cross-modal visual-auditory stimulation, the bilateral activation of V1 was accompanied by a bilateral activation of the perisylvian area corresponding to the auditory primary cortex, and more specifically to the left (BA 41; number of clustered vertices = 20; peak maximum x = −46.84, y = −37.81, z = 16.29; t>5) and right Heschl’s Gyrus (BA 41; number of clustered vertices = 19; x = 51.07, y = −36.95, z = 19 t>5).

### ISI-related Activity

The sLORETA also individuated a bilateral activation of a restricted frontal cortical region starting by around 1,000 ms from S1 offset and reaching a maximum value at 1,506 ms from S1 offset. More specifically, this area corresponded to the left (BA 6; number of clustered vertices = 40; peak maximum x = 9.9, y = 19.2, z = 59; t>8) and right SMA (BA 6; number of clustered vertices = 34; x = −6.9, y = 19.1, z = 60.1; t>9). The scalp-projection of the vertices showing the maximum peak of activation for the whole SMA corresponded around to the FCz electrode in the 10–20 international system. The SMA showed a time course characterised by a clear inverted, U-shaped pattern, with a maximum at S2 METP. After this point, it decreased slowly until the end of the period for deviant ISI.

## Discussion

The present study aimed at unravelling the contribution of task-related processes, including response preparation, selection and execution, from that more directly linked with timing mechanisms in a temporal expectancy task. To this purpose, a ‘passive temporal oddball task’ was created in which participants were not given any task at all. This task was characterised by a passive over-exposition to a 1,500 ms standard S1-S2 ISI, delivered 70% of the total number of trials. Such probabilistic distribution was used in order to implicitly generate a temporal rule. According to such a rule, the maximum probability of occurrence of a specific event (i.e., the S2 onset) was at a precise time point, here defined as S2 Maximum Expectation Time-Point or S2 METP. We hypothesised that a temporal expectancy-related CNV would occur even in the total absence of any motor request. This component might here directly mirror an automatic and action-independent mechanism consisting in the use of the temporal information to create an expectancy of a given event. We also speculated that if the SMA plays a crucial role in perceptual timing, then this area should stay activated while expecting S2, showing typical time course activation characterised by the peak maximum at S2 METP..

As expected, either for the standard and the two deviant ISI conditions, a clear CNV at central scalp-ROI was elicited with a pattern characterised by a progressive, negative increase starting after the end of a S1-locked complex and peaking at the end of the standard ISI corresponding to S2 METP. Then, in the standard ISI trials a sensory-evoked complex elicited by S2 presentation followed the CNV peak, while in the two deviant ISI trial types it showed a shift towards baseline. The fact that CNV slope inversion for deviant ISIs occurred at S2 METP suggests that participants implicitly learned the temporal rule, and that S2 onset was maximally expected at the same time point, regardless of the actual duration of the ongoing ISI. This hypothesis was further confirmed by the presence of a significant time-on-task effect on the CNV morphological pattern, as it became more negative and steeper block-by-block. This effect might be explained by assuming that participants discovered the temporal structure of the trial across the whole experimental session. Notably, this result seems to be apparently at odd with previous studied demonstrating time-on-task habituation on CNV [39]. However, it is likely that the fact that, since in our paradigm there was uncertainty on the exact moment of S2 onset (given that in 30% of the case it occurred later than expected), this did not let the CNV to habituate trial-by-trial but rather to emerge becoming steeper and larger as the temporal structure was progressively and automatically discovered.

The statistical analyses confirmed that the CNV amplitude did not differ between ISI conditions in the interval preceding S2 METP (TW-1). By contrast, after S2 METP ms (TW-2) only for the 1500-Standard ISI condition, the CNV showed a positive voltage while it was still negative for the two deviant ISIs. This last finding was clearly due to the S2-evoked activity. The slope analysis confirmed that the CNV pattern was characterised by a negative trend until S2 METP for all ISI conditions that shifted toward positivity soon after this point. The fact that the CNV showed the same slope and amplitude for all ISIs in TW-1 was an expected finding, easily accounted by considering that until S2 METP participants could not know which trial condition they were looking at, then how long they had to wait before seeing S2. By contrast, the fact that the CNV presented no differences in amplitude but an inverted positive slope in TW-2 (except for the standard ISI condition, see above) was a confirmation that, no matter how long the actual S1–S2 ISI was, once the participants became automatically attuned to the standard ISI S2 was maximally attended at the end of such duration. Therefore, what we finally observed was that even if participants did not overtly estimate time passage and although they were given any task, they nevertheless showed an automatic expectancy based on the capacity to process time by exploiting some internal timer mechanism.

A similar CNV time-course pattern has already been reported in studies using explicit discrimination tasks [30]–[35] and showing a typical shifting in slope at a point in time in which the comparison with the stored reference duration has ended. However, all these studies required response selection between alternatives, leaving unanswered whether the CNV could reflect additional cognitive mechanisms other than perceptual timing. Concerning implicit timing tasks, it is important to mention earlier studies using oddball-like stimulation protocols to investigate timing or time-related processing [40], [57]–[60]. As far as we know, however, no studies used a passive task to elicit temporal CNV by simply manipulating the probabilistic distribution of the ISI between pairs of stimuli. Furthermore, to our knowledge this is the first study that investigated the slow ERP activity during the ISI itself, while most of the studies using passive paradigm focused on stimulus-locked ERP responses, such as the duration-change Mismatch Negativity (MMN) response [59]–[63].

As an important consideration about the protocol we used, however, it should be recognised that although based on a temporal manipulation, the S2 onset expectancy-related brain activity could to some extent be affected by the probability-manipulation per se rather than by timing. This issue assumes importance in relation to previous literature that has shown the CNV to be sensitive to both the duration of ISI and to the probability of occurrence of a target stimulus [64]–[66]. This is a concern intrinsic to all experimental procedures based on passive habituation to a given frequent event (i.e., in the present case the standard ISI). Nevertheless, our CNV results are essentially in line with a temporal explicative account, since: 1) although based on a oddball probabilistic distribution with a condition being highly more frequent that the others, the only variable that was here manipulated was the temporal distance between S1 and S2 (ISI), while these last two never changed; 2) the timing of the shift in polarity of the CNV clearly suggests that the expectation of stimulus appearance was based on a memorized standard duration, therefore, we talk about a ‘temporal’ expectation; 3) the CNV pattern mirrors previously observed findings on timing [28], [30]–[35]. This evidence is in line with the hypothesis that regular target presentation leads to a memory formation of the target-to-target interval, and that such memory trace might contribute to the generation of a time-based expectancy that in turn elicits a CNV developing before target presentation [14]. Our findings further show that this mechanism is automatically engaged whenever a sensory environment is statistically structured so that an exogenous expectation of a given event may be built-up according to a given temporal rule.

Moreover, our data also provides more insight in the role of CNV in timing. In this regard, one of the main theoretical accounts links this slow potential to a temporal ‘cumulative’ mechanism, consisting of the capacity to receive and store time-unit pulses coming from a pacemaker [30]–[32]. This would make the CNV an ERP marker directly implicated in the automatic representation of the unfolding of time *per se* rather than to other computational stages. Traditionally, the fact that the CNV amplitude correlates with the length of the interval to be timed has been interpreted in favour of this view [31]. It must be noticed, however, that a recent study by Kononowicz et al. [39] did not support this view by failing to find amplitude differences in relation to the reproduction of a 2.5 s duration. Moreover, as above mentioned, the authors found a clear time-on-task decreasing effect in the CNV amplitude, probably due to a habituation effect not compatible with the cumulative interpretation.

The fact that we found a slope difference between TW-1 and TW-2 (from negative to positive) despite no differences in amplitudes supports the interpretation that the temporal CNV does not simply reflect the time values currently stored in the accumulator just as a function of time elapsing. According to the conclusions of Kononowicz et al. [37], [39], an alternative explanation could be that the CNV reflects the unfolding of time in a more indirect way, for example, being sensitive to the difference between the current time and an earlier memorized standard duration or, as in the present case, between the current time and a standard duration. In this case, the CNV would reflect not just a simple time-units accumulator but also a temporal mnestic comparison mechanism. However, our data do not completely allow us to distinguish between these two processes, and further research is needed to more directly address this issue.

From a theoretical perspective, the results of this study can be interpreted in the light of the Scalar Expectancy Theory [38], [67], which proposes the existence of a common cognitive mechanism that intervenes in all discrete timing operations. Specifically, the SET theory postulates the existence of an ‘internal clock’ marking out time through the combined activity of a pacemaker that emits pulses and an accumulator that collates and integrates such pulses. This mechanism is also known as a pacemaker-accumulator system or PAS [46]. This model also assumes that the clock is the first step in a three-stage process. In the second step (the ‘memory stage’), the pulses accumulated during the currently perceived or produced interval are counted and briefly stored in working memory so that they can be compared with an interval previously stored in the reference memory. In the third and final stage (the ‘decision stage’), a decision is made on the basis of a comparison between the duration stored in the form of discrete temporal units in the reference memory and the currently perceived duration.

In line with the SET model [38], our data suggest that the pacemaker and comparison stages are dissociable from explicitly-driven decision, such as selecting between alternatives, challenging the idea that CNV peak may necessarily correlate with a time-based overt decisional process [32]. Our results suggest that the brain could actually and automatically compare a deviant ISI to a standard one, even without the involvement of an explicit decision process. Indeed, whether some sort of unconscious internal decisional or ‘categorisation’ mechanism [36] may have taken place after S2 METP in deviant conditions still remains an open question. As a matter of fact, given the absence of task instructions and behavioural data in our paradigm, there is no way to verify this hypothesis, therefore there is also no way to verify the inverse hypothesis.

### The Neural bases of Automatic Temporal Expectancy

The brain source analysis performed on the S1-related ERP activity identified bilateral and simultaneous activation of both primary visual and auditory cortical areas starting at around 100 ms from S1. This double occipital-temporal activation was due to the fact that S1 was always presented together with a synchronised 1,000-Hz sinusoidal tone, and this was reflected in the activation of a restricted temporal area corresponding to the auditory primary cortex and in particular to the HG. After S1 offset, both visual and auditory cortices did not show any significant activation until S2 onset, whereas a bilateral neural activity started in a restricted cortical area of the frontal lobe, corresponding to the SMA. Importantly, this locus showed increasing time course activation throughout the S1-S2 ISI duration (although reaching statistically significant values after around 1,000 ms from S1 offset) and peaked at S2 METP. Importantly, the ISI-related time course activation of the SMA further suggests that this area may be the main candidate as temporal-CNV generator in automatic temporal expectancy. This finding confirms previous data identifying in the SMA a pivotal neural generator of the temporal CNV and recognising a crucial role of this area for timing in general [24]. After S2 METP, SMA displayed a decrease in activation and a return toward the baseline. The ISI-related time course of SMA activation suggests that such a structure plays a crucial role in implicit perceptual timing processes, and in particular to the automatic use of the temporal information to anticipate specific event occurrence even when this does not involve actions or explicit decision. According to the SET theory, it has been suggested that SMA may be part of a network underpinning the PAS as a part of a cerebral circuit that emits oscillatory pulses from the basal ganglia to the superior cortical layers of the frontal lobe [1], [5], [6]–[9], [21], [42]–[46], [68]. In this sense, the CNV has been suggested to be the projection on the scalp in the form of ‘climbing neuronal activity’ [69]–[71] of the basal ganglia-SMA circuit activation during the encoding of discrete time intervals. An alternative and physiologically more plausible hypothesis about the interaction between the basal ganglia and superior cortical areas known as the Striatal Beat Frequency model or SBF [1], [44]–[46] posits that timing arises as a result of the coincidence in the oscillatory activity of the basal ganglia, prefrontal cortex and cerebellum due to the capacity of spiny neurons to detect patterns of activity in the cortical input vector. In this case, a bi-directional, dynamic and large-scale brain network is assumed to underlie timing rather than a simpler unidirectional firing projection from the basal ganglia to superior cortical layers. Even if definitive evidence for a specific model of brain functioning in interval timing is still lacking, there is a general agreement that both the basal ganglia and prefrontal regions, notably the SMA, play a crucial role in time processing. Indirect confirmation of this can be seen by the fact that patients with Parkinson’s disease, a pathology known to involve striatal dopaminergic neuron depletion, as well as frontal cortical alteration, have been shown to display a different CNV morphological pattern compared to healthy controls in tasks requiring implicit time discrimination [72]. Concerning the role of deeper neural generators, several studies demonstrated the involvement of the basal ganglia and cerebellum in both automatic and controlled timing, and a unified model [8], [73]–[75] of time perception has been recently proposed based on coordinated activity in the core striatal, olivocerebellar and cortical networks [76]. As a matter of fact, however, we can here only suggest the involvement of the subcortical and cerebellar structures in automatic temporal expectancy since the ERP brain-source analysis provides reliable results only for cortical areas.

In addition, our data suggest that, as for the CNV, the function of this area in time processing may be more complex than just accumulating time-units *per se* from a pacemaker. In fact, the SMA pattern we found was not only characterised by a raise in activity due to an increase of the neural firing, but also it displayed a precise time-course pattern mirroring the standard ISI rather than the actual duration of the current interval. Whether SMA may be considered as a ‘core’ structure in temporal processing in general, however, is still far from being demonstrated, since additional studies have to be done to better address this issue. The most plausible hypothesis is that this area is part of a wider, anatomically and functionally integrated neural network including subcortical and thalamic structures, and identified in the dopaminergic striato-frontal pathway, playing a central role for time computational activity in the brain [44]–[46]. Finally, an alternative but not opposite explanation of the CNV and the time-course of SMA activity we reported might rely on a working memory maintenance process automatically engaged by the comparison between the current ISI and the standard one stored in memory [77].

### Conclusions

In this study, we reported an ISI-related electrophysiological marker reflecting automatic temporal expectancy. In particular, as far as we know, this is the first data showing a temporal CNV on the only basis of a passive over-exposition to a specific ISI. Indeed, the use of a passive temporal oddball paradigm, requiring neither time-based motor response nor explicit decision-making, allowed participants to access an automatic cognitive mechanism engaged in the environmental temporal structure decoding. From a theoretical viewpoint, the demonstration of a passive temporal CNV suggests that time comparison can be dissociated from other task-related processes like explicit decision and motor processing. This data ultimately confirms that our cognitive system computes the event temporal structure even when this operation is not finalised to a determinate action or overt decision. This mechanism implies the ability to automatically track the temporal statistical regularities in order to anticipate the onset of events and is associated with the involvement of the SMA as a part of a wider network probably entailing the basal ganglia and cerebellum. From a methodological perspective, this study also demonstrates that it is possible to investigate neural mechanisms underlying temporal expectancy using an experimental paradigm requiring neither behavioural responses nor explicit attention to the task from participants, so it is a reliable method that can be applied in non-collaborative subjects, such as infants or patients with consciousness disorders.

## Supporting Information

Table S1 **CNV amplitude and slope values for single subjects.**
(TIFF)
