# Supplementary figures and images for: Automatic Temporal Expectancy: A High-Density Event-Related Potential Study
Source: PLoS One. 2013 May 1;8(5):e62896. doi: 10.1371/journal.pone.0062896 (PMC3641105; doi:10.1371/journal.pone.0062896)

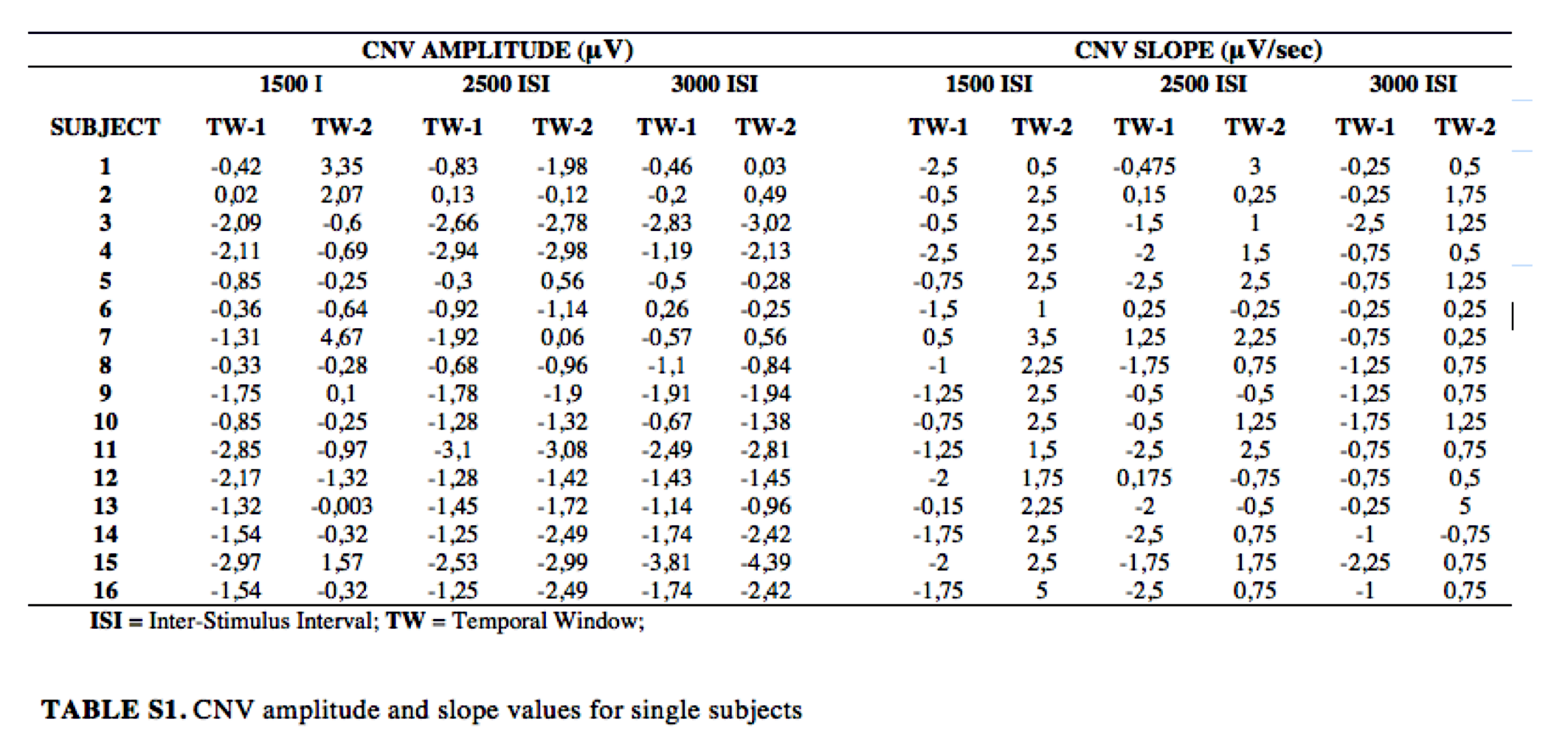

Supplement: Table S1 — CNV amplitude and slope values for single subjects. (TIFF) [file pone.0062896.s001.tiff]
